# Characterisation of an aptamer against the Runt domain of AML1 (RUNX1) by NMR and mutational analyses

**DOI:** 10.1002/2211-5463.12368

**Published:** 2018-01-02

**Authors:** Kenta Takada, Ryo Amano, Yusuke Nomura, Yoichiro Tanaka, Shigeru Sugiyama, Takashi Nagata, Masato Katahira, Yoshikazu Nakamura, Tomoko Kozu, Taiichi Sakamoto

**Affiliations:** ^1^ Department of Life and Environmental Sciences Faculty of Engineering Chiba Institute of Technology Narashino Japan; ^2^ Division of Medical Devices National Institute of Health Sciences Tokyo Japan; ^3^ Facility for RI Research and Education Instrumental Analysis Center Yokohama National University Japan; ^4^ Faculty of Science and Technology Kochi University Japan; ^5^ Institute of Advanced Energy Kyoto University Uji Japan; ^6^ RIBOMIC Inc. Tokyo Japan; ^7^ Department of Basic Medical Sciences Institute of Medical Science University of Tokyo Japan; ^8^ Research Institute for Clinical Oncology Saitama Cancer Center Ina Japan

**Keywords:** AML1, aptamer, Mutation, NMR

## Abstract

Since the invention of systematic evolution of ligands by exponential enrichment, many short oligonucleotides (or aptamers) have been reported that can bind to a wide range of target molecules with high affinity and specificity. Previously, we reported an RNA aptamer that shows high affinity to the Runt domain (RD) of the AML1 protein, a transcription factor with roles in haematopoiesis and immune function. From kinetic and thermodynamic studies, it was suggested that the aptamer recognises a large surface area of the RD, using numerous weak interactions. In this study, we identified the secondary structure by nuclear magnetic resonance spectroscopy and performed a mutational study to reveal the residue critical for binding to the RD. It was suggested that the large contact area was formed by a DNA‐mimicking motif and a multibranched loop, which confers the high affinity and specificity of binding.

**Abbreviations**
AML1: acute myeloid leukaemia 1
HMQC: heteronuclear multiple quantum coherence
ITC: isothermal titration calorimetry
NF‐κB: nuclear factor‐κB
NMR: nuclear magnetic resonance
NOESY: nuclear Overhauser effect spectroscopy
RDE: Runt‐binding double‐stranded DNA element
RD: Runt domain
SELEX: systematic evolution of ligands by exponential enrichment
SPR: surface plasmon resonance

Aptamers are short oligonucleotides that can bind with high affinity and specificity to a wide range of target molecules, which can be generated by an *in vitro* technique known as systematic evolution of ligands by exponential enrichment (SELEX) 1, 2, 3, 4. Recent advances in high‐throughput technology have improved the efficiency of aptamer production 5, 6. Aptamers are expected to be useful as therapeutic agents due to the following characteristics: high affinity and specificity comparable to those of antibodies (*K*_d_ in the nanomolar to picomolar range), moderate molecular mass and ease of chemical production 7, 8, 9.

AML1 (RUNX1) is a transcription factor that plays important roles in maintaining haematopoiesis and immune function in adults 10, 11, 12. AML1 contains a DNA‐binding domain, known as the Runt domain (RD), which recognises a specific DNA element, ‘YGYGGTY’ (where Y = pyrimidine) 13, 14. The tertiary structure of RD has been investigated by X‐ray crystallography and nuclear magnetic resonance (NMR) spectroscopy 15, 16, 17, 18. These studies revealed that RD recognises the DNA element using two loop regions and a C‐terminal tail. Three guanine bases in the major groove of the DNA element are recognised by three arginine residues in the C‐terminal tail and one of the loop regions. The other loop region interacts with the minor groove.

AML1 was originally isolated from a chromosomal break point in a case of human acute leukaemia 19. RNA aptamers that bind to RD were previously studied regarding their potential utility in the diagnosis and treatment of AML1‐related diseases 20, 21, 22, 23, 24. We have already obtained RNA aptamers (Apt1‐S and S4‐S) that show higher affinity (*K*_d_ = 0.99 ± 0.02 nm and 0.034 ± 0.004 nm, respectively) than the Runt‐binding double‐stranded DNA element (RDE, *K*_d_ = 9.6 ±0.2 nm) 21, 22. Structural study of Apt1‐S using NMR revealed that it contains a DNA‐mimicking motif, which adopts a B‐type DNA‐like conformation 23, 25. Furthermore, kinetic and thermodynamic studies of S4‐S using surface plasmon resonance (SPR) and isothermal titration calorimetry (ITC) revealed that S4‐S recognises a large surface area of RD 22. In this study, we performed NMR and functional mutation studies of S4‐SS, which was designed for such studies, and revealed that the aptamer binds to RD using a large contact area, which is consistent with the findings of our previous thermodynamic study.

## Materials and methods

### Expression and purification of AML1 RD and its mutants

AML1 N‐terminal fragment (amino acids 1–188, referred to as the RD) and its mutants were prepared as described previously 21, 22. The purified proteins were dialysed against buffer [20 mm sodium phosphate (pH 6.5), 2 mm magnesium acetate, 300 mm potassium acetate, 50% glycerol and 1 mm DTT] and stored at −25 °C. The concentrations of proteins were determined based on the molecular absorption coefficient at 280 nm.

### Aptamer preparation

S4‐SS and its mutants were synthesised by *in vitro* transcription as described previously 22. The template of Apt1‐S was purchased from Hokkaido System Science Co., Ltd (Sapporo, Japan) and amplified by PCR. 5′‐(T)16‐Primer was used to attach an A16‐tag for use in SPR assays.

### SPR assays

SPR assays were performed as described previously 22 using a BIAcore X instrument (GE Healthcare, Sunnyvale, CA, USA). A Langmuir (1 : 1) binding model was used to analyse the association rate constant *k*_on_ and the dissociation rate constant *k*_off_. The dissociation constant *K*_d_ was also determined as the ratio of *k*_off_ to *k*_on_, and is presented as the mean ± standard error of three independent measurements.

### NMR measurements

The RNA samples were annealed by heating at 95 °C for 5 min followed by snap cooling on ice. Purified S4‐SS and ^15^N‐labelled S4‐SS were dissolved in 20 mm sodium phosphate (pH 6.5). Concentrations of S4‐SS and ^15^N‐labelled S4‐SS were 0.4 and 0.02 mm, respectively. NMR spectra were measured using an Avance600 spectrometer (Bruker BioSpin, Billerica, MA, USA). Spectra were recorded at a probe temperature of 10 °C. The imino proton resonances of G and U residues were distinguished by the ^1^H‐^15^N heteronuclear multiple quantum coherence (HMQC) spectrum measured with ^15^N‐labelled S4‐SS 26. Exchangeable proton resonances were assigned by nuclear Overhauser effect spectroscopy (NOESY) in H_2_O with a mixing time of 150 ms using the jump‐and‐return scheme for water suppression 27.

## Results

### Truncation of aptamer for NMR analysis

**Figure 1:**
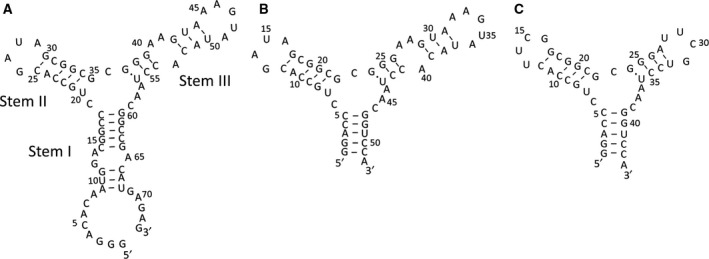
Design of S4‐SS for NMR and mutational studies. Predicted secondary structures of S4 (A), S4‐S (B) and S4‐SS (C). The 5′‐end and 3′‐end of stem I were truncated for S4‐S. The GAUA loop of stem loop II was replaced by UUCG tetraloop, and stem loop III was shortened and capped by UUCG tetraloop for S4‐SS.

We had already constructed S4‐S, which comprises two stem loops (stem II and III) and one multibranched loop from the 17th to 61st nucleotide of S4, the 5′‐GGA for effective transcription initiation by T7 RNA polymerase, and a 3′‐UCCA for stabilising the stem I structure, resulting in a length of 52 nucleotides (Fig. 1A, B) 22. For functional mutation study of the aptamer, it is useful to stabilise the conformation of the aptamer because mutations may sometimes induce large conformational change. It is difficult to judge whether the mutated residue is involved in direct interaction or important for folding of the active conformation, if conformational change is easily induced by a mutation. Thus, we designed S4‐SS (44 nucleotides), in which GAUA of stem loop II is replaced by a stable UUCG tetraloop and stem loop III is shortened and also capped by a stable UUCG tetraloop (Fig. 1C). As the UUCG tetraloop has been well characterised by NMR 28, 29, substitution of the stem loops by the tetraloop helped us analyse the NMR spectra of the aptamer.

The apparent dissociation constant (*K*_d_) of S4‐SS for RD binding, calculated from the SPR profiles, was 0.289 ± 0.006 nm, whereas those of S4 and S4‐S were 0.044 ± 0.002 and 0.034 ± 0.004 nm, respectively (Table 1). Substitution of the two stem loops by the UUCG tetraloop had limited effects on binding affinity.

**Table 1:** Binding affinities of S4 mutants to the Runt domain

Substitution	*k*_on_ (m ^−1^·s^−1^) × 10^6^ a	*k*_off_ (s^−1^) × 10^−3^ a	*K*_d_ (nm)^a^	Relative affinity^b^
S4^c^	9.1 ± 0.1	0.40 ± 0.02	0.044 ± 0.002	6.6
S4‐S^c^	10.7 ± 0.3	0.37 ± 0.06	0.034 ± 0.004	8.5
S4‐SS	8.6 ± 0.5	2.5 ± 0.2	0.289 ± 0.006	1
C5GG39C	10.2 ± 0.4	1.8 ± 0.2	0.18 ± 0.02	1.6
C6A	–	–	> 1.0 × 10^3^ d	< 3 × 10^−4^
U7C	1.03 ± 0.06	37 ± 4	35 ± 3	0.008
G8CC21G	9.6 ± 0.7	9.9 ± 0.5	1.04 ± 0.09	0.28
C9GG20C	–	–	> 1.0 × 10^3^	< 3 × 10^−4^
C10GG19C	–	–	> 1.0 × 10^3^	< 3 × 10^−4^
A11G	8.9 ± 0.2	24 ± 1	2.7 ± 0.1	0.11
C12GG17C	7.4 ± 0.3	37 ± 1	4.94 ± 0.01	0.06
G22A	7.6 ± 0.8	37 ± 4	4.8 ± 0.2	0.06
C23A	13 ± 1	5.2 ± 0.1	0.39 ± 0.02	0.74
G24A	12 ± 1	15 ± 1	1.3 ± 0.2	0.22
G25CC34G	10.7 ± 0.5	4.03 ± 0.03	0.38 ± 0.02	0.76
U35C	5.7 ± 0.9	60 ± 8	10.6 ± 0.6	0.03
A36U	10 ± 1	14 ± 2	1.3 ± 0.3	0.22
A37U	–	–	> 1.0 × 10^3^	< 3 × 10^−4^
C38U	–	–	> 1.0 × 10^3^	< 3 × 10^−4^

^a^A Langmuir (1 : 1) binding model was used to analyse the association rate constant, *k*_on,_ and the dissociation rate constant, *k*_off_. The dissociation constant, *K*_d_, was also determined as the ratio of *k*_off_ and *k*_on_ as follows: *K*_d_ = *k*_off_/*k*_on_, and is presented as the mean ± SE (*n* = 3). ^b^Relative affinity was calculated with the affinity of S4‐SS set as 1. ^c^These data are taken from a previous report 22. ^d^ *K*_d_ values were estimated as > 10^3^ nm if the increase in RU was too small to calculate the *K*_d_ values when 1.0 × 10^3^ nm RD was injected.

### Analysis of the secondary structure of S4‐SS by NMR

**Figure 2:**
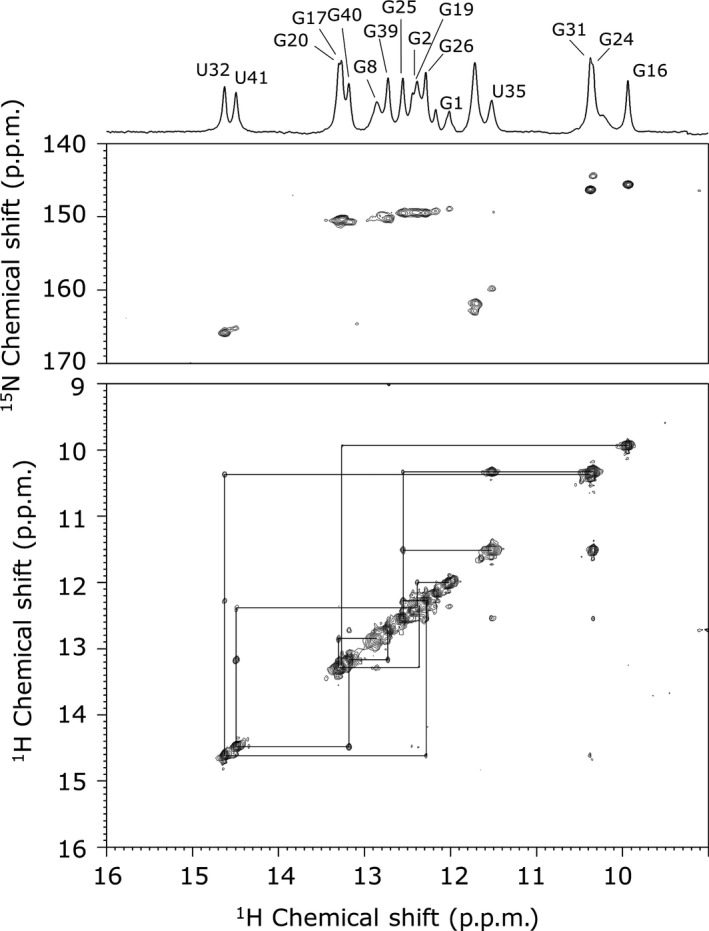
1H‐15N HMQC and NOESY spectra of S4‐SS in H_2_O and assignment of imino proton signals. 1D imino proton spectrum (upper), 1H‐15N HMQC spectrum (middle) and 2D NOESY (mixing time = 150 ms) spectrum (lower) of S4‐SS are shown. NOE connectivities are indicated by lines. Assignments were determined by NOE connectivities for imino proton resonances of G1–G2–U41–G40–G39, G8–G20–G19, G17–G16 and U35–G24–G25–G26–U32–G31, which are shown on top of the 1D imino proton spectrum.

To confirm the secondary structure of S4‐SS, a NOESY spectrum was measured and imino proton signals were assigned (Fig. 2). The assignment of imino proton signals was confirmed using the ^1^H‐^15^N HMQC spectrum. Imino proton signals for guanosine residues of UUCG tetraloops were observed at around 10 ppm, which is a typical value for them 28, 29. NOE connectivities for imino proton resonances of G1–G2–U41–G40–G39, G8–G20–G19, G17–G16 and U35–G24–G25–G26–U32–G31 revealed the formation of three stems, two hairpin loops and one multibranched loop. Furthermore, imino proton signals of the G24–U35 base pair were observed and a typical strong NOE signal between them was observed. However, those of the U7–G22 base pair were not observed, suggesting that the G24–U35 base pair is stably formed, although the U7–G22 base pair is not formed.

### Base specificity of S4‐SS for RD binding

**Figure 3:**
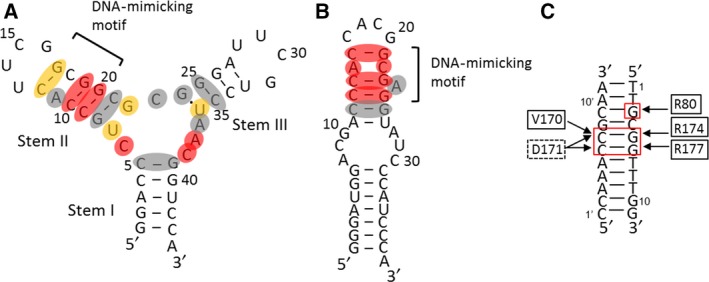
Comparison of mutational analysis of S4‐SS with that of Apt1‐S. (A) Summary of mutational analysis of S4‐SS. The very important residues are coloured red, at which mutation diminished the binding. The moderately important residues are coloured orange, at which mutation decreased the binding from 1/10 to 1/1000 of S4‐SS. The unimportant residues are coloured grey. (B) Effect of mutation on Apt1‐S as reported previously 21. The importance of residues is represented by their colouring, the same as in panel A. (C) RD binding to RDE. The interactions are indicated by arrows, as revealed by the crystal structure 16.

To analyse the base specificity of S4‐SS for RD binding, we performed mutation analysis. Dissociation constants of S4‐SS mutants were studied by SPR (Table 1, Fig. S1). Mutations at two CG base pairs (C9G–G20C and C10G–G19C), C6G, A37U and C38U markedly diminished the binding activity. Thus, it was suggested that these bases directly interact with RD (Fig. 3A; red). Mutations at the C12G–G17C base pair, U7C, G22A and U35C had moderate effects on binding affinity (1/10 > 1/1000 of S4‐SS) (Fig. 3A; orange). However, mutations at three CG base pairs (C5G–G39C, G8C–C21G and G25C–C34G), A11G, C23A, G24A and A36U had almost no effect or little effect on binding affinity (> 1/10 of S4‐SS) (Fig. 3A; grey).

We previously reported that RD binds to the DNA‐mimicking motif of Apt1‐S, in which C13, C14, G21, G23 and G25 correspond to the nucleotides C6′, C7′, G3, G5 and G6, respectively, in RDE (Fig. 3) 21. Although the effect of mutation at the C12G–G17C base pair on S4‐SS binding to RD is smaller than that on Apt1‐S binding, mutations at C9G–G20C and C10G–G19C completely abolished the binding affinity of S4‐SS, as was also the case for Apt1‐S. Thus, it was suggested that stem II of S4‐SS containing C9, C10, G17, G19 and G20 adopts the DNA‐mimicking motif, as was the case for Apt1‐S.

### RD amino acid residues required for S4‐SS binding

Next, we performed mutational analysis of RD to determine whether S4‐SS binds to the same amino acid residues as in the case of Apt1‐S and RDE. The crystal structure of the RD–RDE complex showed that R80, V170, D171, R174 and R177 are involved in the direct interaction with DNA bases as follows: R80 contacts the N7 and O6 atoms of G3; V170 contacts the C5 atom of C7′; D171 contacts the N4 atoms of C7′ and C6′; R174 contacts the N7 and O6 atoms of G5; and R177 contacts the N7 and O6 atoms of G6 (Fig. 3C) 16. Therefore, these five residues were replaced with alanine and the resulting binding affinities of S4‐SS were analysed by SPR (Table 2, Fig. S2).

**Table 2:** Binding affinities *K*_d_ of RD mutants to S4‐SS.^a^

Substitution	S4‐SS	Apt1‐S^b^	RDE^b^
Wild‐type	0.289 ± 0.006	0.99 ± 0.02	9.6 ± 0.2
R80A	9.0	> 1.0 × 10^3^	> 1.0 × 10^3^
V170A	0.3	0.88 ± 0.07	7.2 ± 0.2
D171A	140	> 1.0 × 10^3^	> 1.0 × 10^3^
R174A	> 1.0 × 10^3^ c	> 1.0 × 10^3^	> 1.0 × 10^3^
R177A	6.1	> 1.0 × 10^3^	60 ± 7

^a^A Langmuir (1 : 1) binding model was used to analyse the dissociation constant, *K*_d_. ^b^These data are taken from a previous report 21. ^c^ *K*_d_ values were estimated as > 10^3^ nm if the increase in RU was too small to calculate the *K*_d_ values when 1.0 × 10^3^ nm RD mutant was injected.

Alanine substitution at either D171 or R174 significantly reduced the binding to S4‐SS, whereas the V170 mutant showed no marked change in binding, which is similar to the case of Apt1‐S and RDE. On the contrary, mutations at R80 and R177 had moderate effects on the binding affinity to S4‐SS, whereas these mutations markedly diminished the binding affinity to Apt1‐S. Therefore, the binding of S4‐SS to RD differed slightly from that of Apt1‐S, although there was basic similarity between them.

## Discussion

Secondary structure analysis of S4‐SS by NMR showed the formation of three stems (stems I, II and III), two hairpin loops and one multibranched loop. Mutational analysis of the aptamer revealed that the three CG base pairs (C9–G20, C10–G19 and C12–G17) in stem II are important for RD binding, although the mutation at the C12–G17 base pair had moderate effects on binding affinity (Table 1). A comparison of the secondary structures of S4‐SS, Apt1‐S and RDE suggested that the nucleotides whose bases are recognised by the RD in S4‐SS, C9, C10, G17, G19 and G20 correspond to nucleotides C13, C14, G21, G23 and G25 in Apt1‐S or C6′, C7′, G3, G5 and G6 in RDE, respectively (Fig. 3). Therefore, it was suggested that these nucleotides in S4‐SS constitute the DNA‐mimicking motif similar to the case of Apt1‐S.

Although S4‐SS seems to have the DNA‐mimicking motif, the degree of importance of the motif in S4‐SS differs from those in Apt1‐S and RDE. Mutations at C12–G17 in S4‐SS (C16–G21 in Apt1‐S; C9′‐G3 in RDE) and R80, which contact each other directly, showed complete loss of binding activity in the case of Apt1‐S and RDE 21; however, they showed limited effects on S4‐SS binding. Furthermore, C6, A37 and C38 are more important than C12–G17 for aptamer binding. Thus, it was suggested that the base specificity of S4‐SS is slightly different from that of Apt1‐S or RDE.

The intensive analyses of an RNA aptamer against the NF‐κB p50 homodimer (p50)_2_ revealed that the aptamer binds to the DNA‐binding site of a transcription factor by mimicking DNA 30, 31, 32. The RNA aptamer against (p50)_2_ had no resemblance to the target DNA in terms of sequence and secondary structure. However, comparison of crystal structures revealed that a DNA guanine recognised by NF‐κB p50 is replaced by two uracils in the NF‐κB p50–aptamer complex. Thus, the aptamer mimics the target DNA elements with its tertiary structure, but the base specificity is different from that of DNA. The knowledge obtained from studies of NF‐κB p50–aptamer and our current results that base specificity differs slightly from that of Apt1‐S and RDE suggested to us that we can obtain aptamers that bind to RD without a DNA‐mimicking sequence by carrying out SELEX using different conditions, primers and so on.

Gelinas *et al*. 33 analysed the crystal structures of aptamer–protein complexes and showed that the sizes of the interaction surface area between aptamers and proteins cover a wider range (348–2599 Å^2^) than those between antibodies and proteins (560–1300 Å^2^). However, binding affinity is not necessarily correlated with the size of the binding surface. They analysed the shape complementarity index (*S*_C_) 34 and suggested that shape complementarity is also important for binding affinity, which is accomplished by the structural plasticity of RNA aptamers, having a high degree of torsional flexibility. In our previous kinetic and thermodynamic studies using SPR and ITC, we proposed that S4‐S binds to RD with long‐range electrostatic force in the early stage of the association and then S4‐S changes its conformation and recognises the large surface area of RD by optimal hydrogen bonding, van der Waals contact and/or hydrophobic interaction 22. Combining the mutation data with previous data, we assume that S4‐S recognises the large surface area of RD by DNA‐mimicking motif and the multibranched loop.

In summary, NMR and mutational analyses have shown the binding properties of S4‐SS binding to RD. We revealed that a high affinity of S4‐SS to RD is achieved by the multiple contacts of DNA‐mimicking motif and multibranched loop. Our results including those described in previous reports may be useful for the rational design of aptamers against many other proteins.

## Author contributions

RA, KT and TS conceived and designed the experiments; KT and RA performed the experiments and analysed the data; Y Nomura, YT, SS, TN, MK, Y Nakamura and TK contributed reagents/materials/analysis tools; TS, RA and TN wrote the manuscript.

## Supporting information


**Fig. S1.** SPR analysis of S4‐SS mutants binding to RD.
**Fig. S2.** SPR analysis of S4‐SS binding to RD mutants.
