# Supplementary material for: Characterisation of an aptamer against the Runt domain of AML1 (RUNX1) by NMR and mutational analyses
Source: FEBS Open Bio. 2018 Jan 2;8(2):264–70. doi: 10.1002/2211-5463.12368 (PMC5794459; doi:10.1002/2211-5463.12368)
Supplement: Supplementary file 1 — Fig. S1. SPR analysis of S4‐SS mutants binding to RD. Fig. S2. SPR analysis of S4‐SS binding to RD mutants. [file FEB4-8-264-s001.pdf]

Supplementary Figures

Characterization of an aptamer against Runt domain of AML1 (RUNX1) by NMR and mutational analyses

Kenta Takada[1,#], Ryo Amano[1, #], Yusuke Nomura[2], Yoichiro Tanaka[3], Shigeru Sugiyama[4], Takashi Nagata[5], Masato Katahira[5], Yoshikazu Nakamura[6,7], Tomoko Kozu[8], and Taiichi Sakamoto[1]

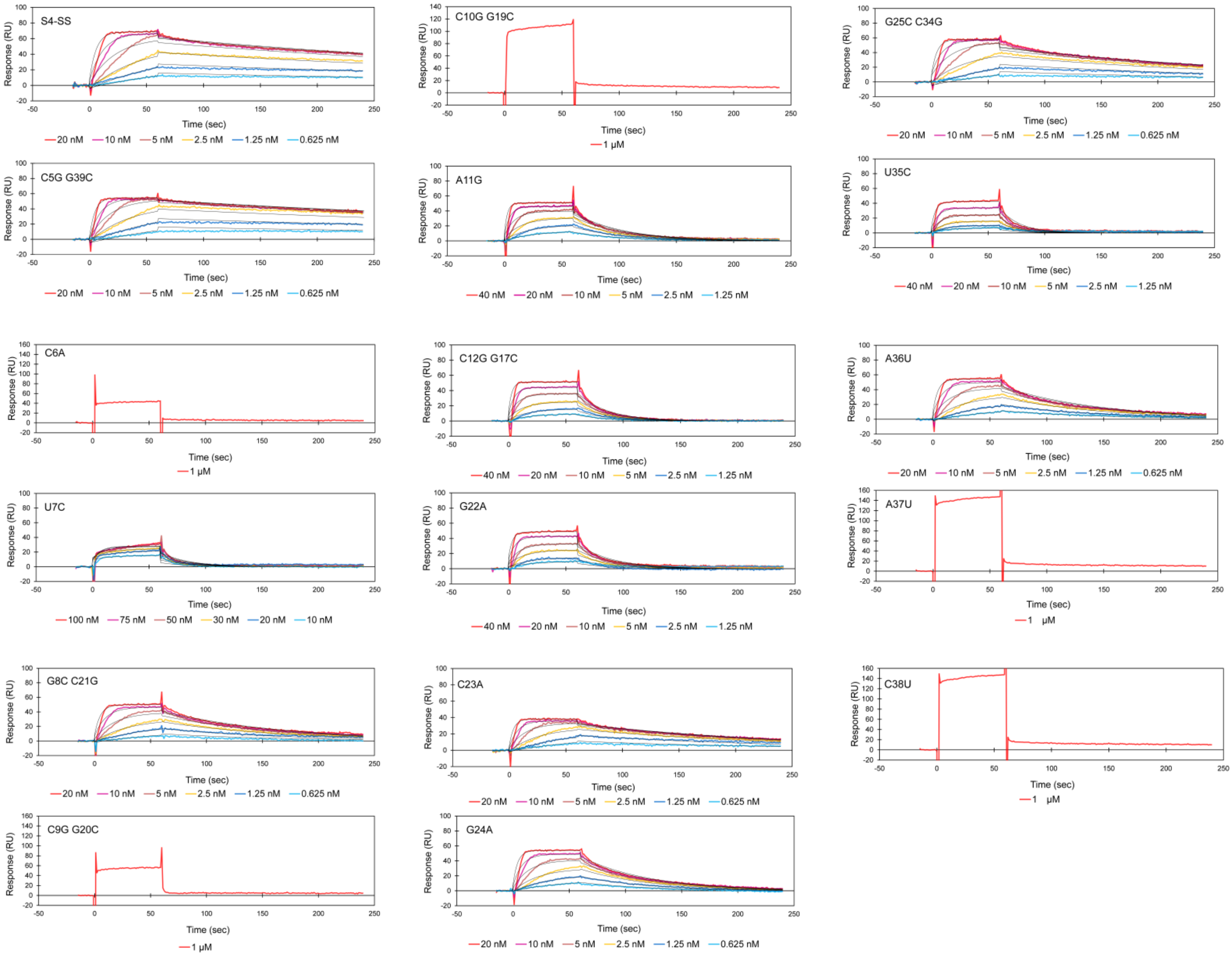

Fig. S1. SPR analysis of S4-SS mutants binding to RD.

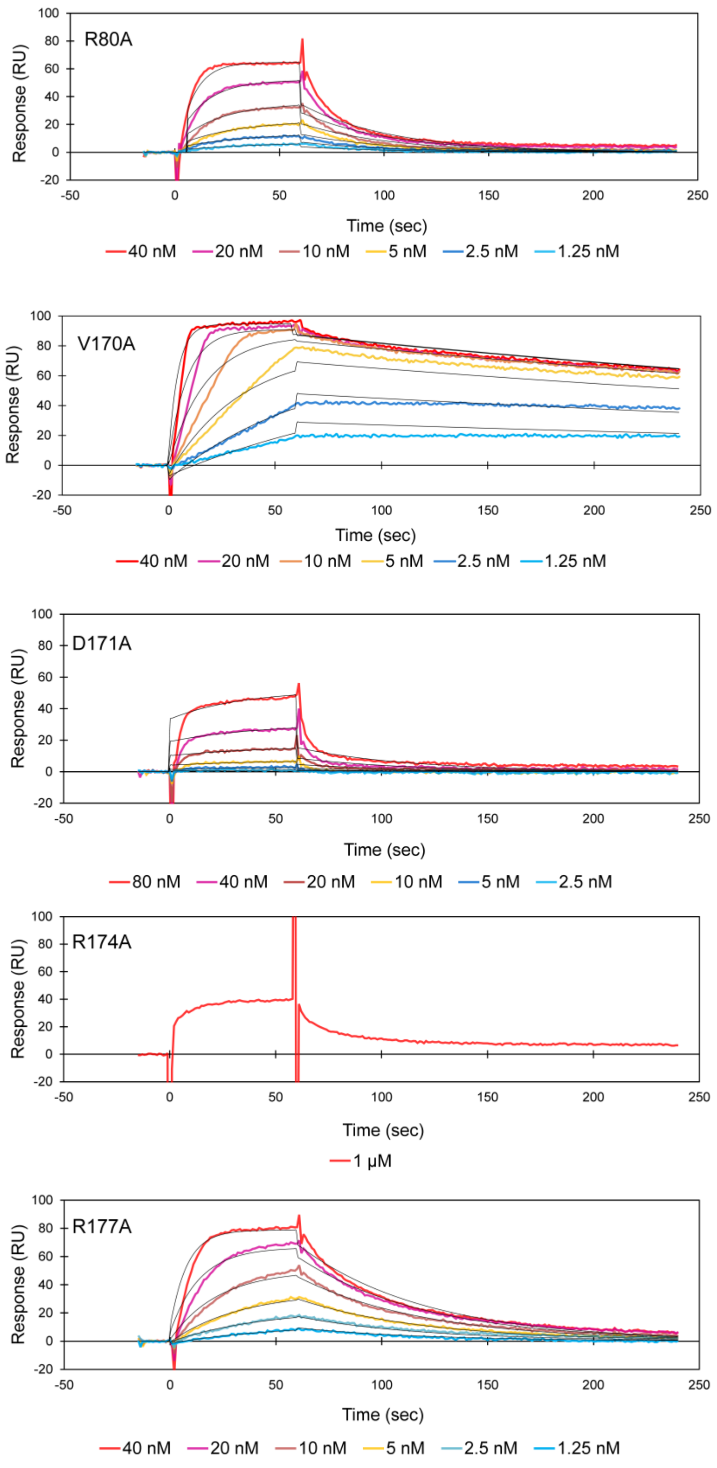

Fig. S2. SPR analysis of S4-SS binding to RD mutants.
